# The role of risk perception and affect in predicting support for conservation policy under rapid ecosystem change

**DOI:** 10.1111/csp2.316

**Published:** 2020-11-11

**Authors:** Freya A. V. St John, Tom H. E. Mason, Nils Bunnefeld

**Affiliations:** 1 School of Natural Sciences, Bangor University, Bangor, UK; 2 Conservation Ecology Group, Department of Biosciences, Durham University, Durham, UK; 3 Biological and Environmental Sciences, University of Stirling, Stirling, UK

**Keywords:** affect, arousal, barnacle geese, conflict, culling, decision-making, emotion, hazard acceptance, psychology, risk, valence

## Abstract

Conservation conflicts are damaging for humans and wildlife, with differences in people's objectives fuelling challenges of managing complex, dynamic systems. We investigate the relative importance of economic, psychological (affect, trust and risk perception) and ecological factors in determining farmers' management preferences, using Greenland barnacle geese (*Branta leucopsis*) on Islay, Scotland, as a case study. Barnacle geese reduce agricultural productivity on Islay, negatively impacting household economies. Since 1992, farmers have received partial compensation but a new culling scheme has escalated conflict between conservation and agricultural interests. Using a questionnaire, we collected data from 75% of the farmers receiving goose payments. We found that affect was a strong driver of both risk perception and management preferences. However, we revealed complexity in these relationships, with trust and economic factors also influencing decision-making. Psychological and economic factors surrounding wildlife management must be understood if we are to achieve conservation objectives in human dominated landscapes.

## 1 Introduction

Differences in stakeholder values are at the heart of conservation conflicts, with dissimilarities in objectives fueling the challenges of managing complex, dynamic systems (Mason, Keane, Redpath, & Bunnefeld, 2018; Redpath, Gutiérrez, Wood, & Young, 2015). The search for sustainable solutions demands integration of ecological and social sciences (Young et al., 2016). In particular, psychological work on judgment and decision-making, risk perception and hazard acceptance (Bruskotter & Wilson, 2014; Gore et al., 2009; Zajac, Bruskotter, Wilson, & Prange, 2012) offers potential for understanding stakeholders impacted by wildlife and the degree to which they want risk reduced. In this paper, we explore relationships between affect, trust, risk perception and management preferences in a dynamic, uncertain system of conservation conflict.

Until recently, virtually all theories of decision-making under risk or uncertainty assumed people cognitively weighed up relative costs and benefits of actions in order to arrive at rational decisions that maximize their utility (Loewenstein, Weber, Hsee, & Welch, 2001). This mode of thinking underpins the expected-utility model that has informed theories of economists and psychologists alike (Leiserowitz, 2006). However, weighing up the utility of every action is cognitively burdensome so people frequently depend upon decision-making shortcuts or “heuristics.” A growing body of literature from several fields has provided compelling evidence that two parallel interacting modes of information processing exist (Epstein, 1994). Dual process models divide human thought processes into two intertwined systems, the experiential and the analytical. The experiential is affective, fast-acting and automatic in nature while the analytical operates more slowly and encompasses deliberative, logical thought processes (Evans, 2003).

Compelling evidence of how affect influences human deliberation in a conservation context was provided by Wilson (2008) who showed how emotion and affect sway individual choice behavior during environmental decision-making. Participants allocated greater funds to affect-rich problems (e.g., petty crime) which posed limited risk to management objectives compared to the affect-neutral issue of deer-overpopulation which constituted a greater environmental risk (Wilson, 2008). The role of the experiential and analytical systems has also been assessed with respect to choices people make to support or oppose wolf-recovery policies in the USA. Findings highlighted the prominence of affect, above more analytical thought processes (Slagle, Bruskotter, & Wilson, 2012). Further, Baynham-Herd, Redpath, Bunnefeld, and Keane (2020) suggest that fast, intuitive thinking may underpin choice of management action for conservation conflicts.

Drawing on psychological theory of risk and hazard acceptance, Zajac et al. (2012) developed a model of wildlife acceptance that incorporated social trust. Theoretically, trust in those responsible for managing hazards decreases perceived risks and ultimately increases acceptability of hazards (Siegrist & Cvetkovich, 2000). Investigating public acceptance of black bears in Ohio, Zajac et al. (2012) found that low levels of trust in the wildlife division were indicative of high risk perception and vice versa. Building upon such work, Bruskotter and Wilson (2014) proposed a hazard-acceptance model specifically for large carnivores which incorporates the roles of trust and affect. To date, such research has focused on carnivores which pose potential risks to livelihoods and human safety. However, in order to develop a more holistic understanding of environmental risk, it is important to work also on species that create less extreme positions.

In this study, we investigate the relative importance of economic, psychological and ecological factors in determining farmers' risk perception and management preferences in a conservation conflict. We use Greenland barnacle geese (*Branta leucopsis*) on Islay, Scotland as a case study because, similar to many other species (especially large grazing birds, ungulates and carnivores), the barnacle goose population has increased rapidly over the last two decades from around 20,000 individuals in 1987 to about 37,500 in 2014 (Scottish Natural Heritage, 2014, 2015). Due to their reliance on a few sites situated on migratory routes, populations of migratory waterbirds are considered to be of high conservation priority (Kirby et al., 2008). Indeed, the Greenland barnacle goose is an Annex I species on the European Union Birds Directive. Supporting around half of the world's population outside of the breeding season, Islay is an important site for this species (Mitchell & Hall, 2020). Having departed breeding grounds in Greenland, geese arrive on Islay, via staging grounds in Iceland, in early October and leave around mid-April (Mason, Keane, et al., 2018). While on Islay, in addition to utilizing natural habitats, the geese feed increasingly on agricultural land — specifically improved grassland — with large flocks substantially impacting grass yields (Percival & Houston, 1992) and imposing a risk to the household economy and well-being of Islay's farmers. To date, management of goose conservation-agriculture conflicts on Islay and elsewhere have generally aimed to reduce damage caused by geese using a multi-pronged approach combining habitat management of goose refuges, scaring of geese from farmland and payment of compensation to farmers experiencing crop and grass damage (Fox, Elmberg, Tombre, & Hessel, 2017). However, where goose numbers continue to increase, their economic impacts can exceed the level of funding for compensation, necessitating population regulation through hunting or culling, such as is practiced on Islay (McKenzie & Shaw, 2017).

Conservation conflict cannot be understood from a single paradigm (Redpath et al., 2013). While ecological processes leading to the emergence of conflict between conservation and agricultural interests on Islay, and more widely across Europe, are understood (Cusack et al., 2019; Mason, Keane, et al., 2018), the psychological dimension of this wildlife-agriculture conflict has not been studied. A lack of understanding of how farmers on Islay perceive “the goose issue” is constraining intervention effectiveness. Compensation schemes designed to mitigate the impacts of conservation conflicts typically seek to redress the economic impact of a conserved species (e.g., paying compensation to owners of livestock killed by carnivores). Put another way, such schemes take a “rational” approach to addressing damage incurred. In designing such schemes, conservationists typically draw on information regarding the ecology of the species and the economic impact of the damage incurred. However, in their design, such schemes ignore a complex array of psychological factors, such as affect and social trust in authorities, known to guide judgments and decisions including those concerning hazards (Siegrist & Cvetkovich, 2000; Slovic, Finucane, Peters, & MacGregor, 2007). In this study we aimed to: (a) better understand the relative importance of ecological (farm-level goose density, distance to roosting site and area of improved grassland), psychological (affect and social trust) and economic (farm and conservation incomes) factors in determining the degree of risk farmers perceive geese impose on their livelihood and household well-being; and (b) to understand the relative influence of these same factors, plus risk perception, on farmer's management preferences for barnacle geese (level of goose harvest and goose-related damage reduction wanted). Based on the theoretical premise that affect has primacy over more cognitive processes (Slagle et al., 2012; Slovic et al., 2007; Zajac et al., 2012), we expected affect — a hereto largely ignored influence — to be more strongly related than other variables to both goose-related risk perceptions and management preferences. By integrating conceptual approaches from natural and social sciences we strive to better understand the human dimensions of this sustained conflict, and conservation conflicts more broadly.

## 2 Methods

### 2.1 Study area

Islay is a 62,000 ha island of the Inner Hebrides, Scotland. Agriculture dominates the landscape (56,000 ha), mostly in the form of rough grazing and improved grassland supporting livestock, but including barley utilized by whiskey distilleries. The 2011 census reported 3,228 people living on Islay; ~18% of those aged 16–74 were employed in the agriculture sector (National Records of Scotland, 2018).

In winter, Islay provides vital habitats for barnacle geese (BG) and Greenland white-fronted (WF) geese (*Anser albifrons flavirostris*), both protected by European law. The BG population on Islay has grown from ~20,000 individuals in 1987 to ~37,500 in 2014. The WF has declined from ~13,000 in the early 1990s to ~4,500 in 2014 (Scottish Natural Heritage, 2014, 2015). Generally, BG feed on improved grassland grown for livestock production. In large numbers, they reduce system-productivity, impacting household economies and the agricultural economy of Islay. Since 1992, farmers have received partial compensation for these losses with farm-level conservation payments taking into consideration the area of improved grassland and quantity of BG observed per farm during annual goose counts. In 2014, a new management strategy was developed by Scottish Natural Heritage (SNH) to meet the UK's EU conservation obligations for geese, minimize economic losses to farmers, and maximize public value for money. The 2014–2024 scheme aims to reduce goose damage on farms by 25–35% by culling 25–30% of BG over a 10-year period (McKenzie & Shaw, 2017). Since 2014, culls on Islay have taken 1,000–2,700 BG annually. The scheme has escalated conflict between farmers and their union (National Farmers Union, NFU), SNH, and conservation organizations including the Royal Society for the Protection of Birds (RSPB) and the Wildfowl and Wetland Trust (WWT). In 2015, RSPB and WWT lodged a formal complaint concerning BG culling to the European Commission (Mason, Keane, et al., 2018), the Scottish Government recognizes the conflict (Scottish Parliament, 2019) but the complaint has not been upheld.

### 2.2 Questionnaire design and administration

We designed a questionnaire (Supporting Information) to measure perceived risks and benefits of different items, affective responses to stimuli, levels of trust in relevant organizations, and degree of support for the 2014–2024 goose scheme. Data on farmer demographics including the percentage of household income derived from on-and off-farm activities and conservation payments were also collected from respondents. Using a seven-point scale, farmers evaluated six items (fertilizers, cigarette smoking, BG, pesticides, vaccinations and WF) according to perceived risk. Then, separated by two questions regarding where funds for goose payments should come from, perceived benefits. Farmers were asked to think of risks/benefits broadly with respect to their livelihoods and household well-being. Measuring perceived risks and benefits of some lifestyle/livelihood activities provides context and reference values from the classic psychological literature against which the relative risk and benefits of geese can be evaluated.

We measured affect on two dimensions, valence and arousal, using two nine-point self-assessment manikin scales (Bradley & Lang, 1994). Each extreme of the valence scale displayed three words: Happy, Pleased and Satisfied matched with Unhappy, Annoyed and Unsatisfied. Calm and Relaxed, Agitated and Irritated denoted the two extremes of the arousal scale. Valence and arousal were coded from one to nine with nine representing a high state of pleasure or agitation, respectively (Lang, Bradley, & Cuthbert, 2008). Following an explanation of the scales, farmers rated a practice image. Time pressure (6 s) was applied to reduce analytical deliberation and increase salience of affective processes (Finucane, Alhakami, Slovic, & Johnson, 2000). Each BG or WF image (Table S1) was interspersed with an image from the International Affective Picture System library (Lang et al., 2008) to maintain participant engagement (Table S2). Assessment of BG and WF images were separated by questions on trust. National and local branches of three organizations, NFU, RSPB and SNH, were assessed (1 = No trust at all; 7 = Trust completely).

Support (1 = Very unsupportive; 5 = Strongly supportive) for the BG scheme was measured by presenting farmers with three scenarios. The first described the current scheme: *Over a ten-year period, goose damage will be reduced by 25–35%. This will be achieved by shooting 25–30% of the barnacle geese on Islay over 10 years. Goose payments will remain the same. Subsidies and average prices of agricultural goods* (*both purchased and sold*) *will remain at today's levels, although some small variation can be expected between years*. The second allowed farmers to adjust the numeric values included in scenario one. The third was identical to the first but stated that due to funding cuts, BG payments would be halved.

The questionnaire was refined through discussions with SNH before piloting. Minor revisions were required post-pilot, so we excluded pilot data from analyses. Given only 81 farmers registered for the goose scheme in 2016, we attempted to survey them all. Appointments were made in advance of FSJ visiting farmers. Free prior informed consent was always acquired and anonymity was guaranteed. University of Kent provided ethical approval.

### 2.3 Ecological data

**Figure 1:**
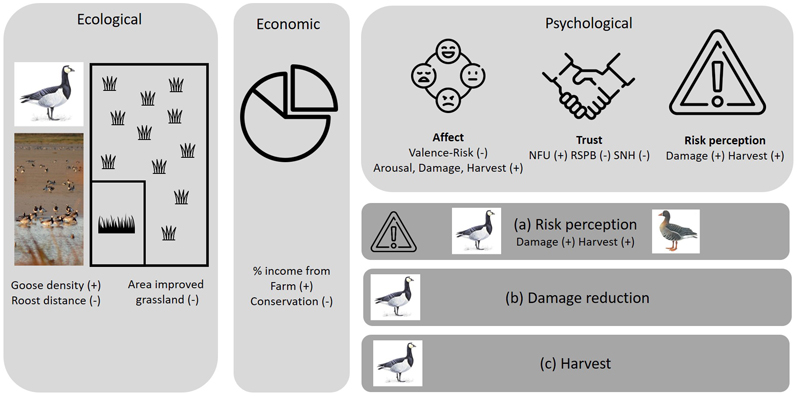
Conceptual framework indicating direction of hypothesized relationships between ecological, economic, and psychological factors and (a) risk perception of barnacle or white fronted geese; (b) desired level of BG damage reduction; and (c) preferred level of barnacle goose harvest. Unless specified, direction of hypothesized relationship is identical across all three response variables. Icons made by Freepik from www.flaticon.com. Goose images used with permission from the RSPB (Mike Langman [rspb-images.com])

Farm-specific data on goose density, distance to roost and area of improved grassland were incorporated into our analyses to investigate relationships between ecological conditions and farmers' perceptions. Farm-specific goose density per hectare, for BG and WF, was calculated using goose survey data from SNH for 1998–2016 (Mason, Keane, et al., 2018). Islay hosts three night-time BG roosts; we calculated the Euclidean distance between participants' farms and the nearest roost. We calculated the farm-specific area of improved grassland (reseeded in preceding seven years) using goose scheme data (Mason, Keane, et al., 2018). We hypothesized valence (Bradley & Lang, 1994), trust in Royal Society for the Protection of Birds (RSPB) and Scottish Natural Heritage (SNH), proportion of income derived from conservation payments, area of improved grassland and distance to nearest roost to be negatively related to risk and the severity of management farmers desired. Further, we expected arousal (Bradley & Lang, 1994), trust in the National Farmers Union (NFU), farm-level barnacle goose density and the proportion of household income derived from the farm to be positively related to risk and the level of goose management farmers wanted to see (Figure 1).

### 2.4 Data analysis

We completed analyses in R version 3.61 (R Core Team, 2019) and IBM-SPSS Statistics 24. Data were checked for normality (Kolmogorov–Smirnov test and checking Q-Q plots). Relationships between perceived risks and benefits, valence and arousal were investigated using Spearman's Rank correlation coefficients. The Wilcoxon test was used to examine within-respondent differences in levels of trust (national:local) and support for management (scenario 1:scenario 3). Assessments of risks and benefits become confounded in the mind and if all farmers perceived items assessed as high risk to be of low benefit, the risk–benefit correlation could be zero despite discrepancies in risk–benefit perceptions (Alhakami & Slovic, 1994). For this reason, following Alhakami and Slovic (1994) we calculated the absolute difference, hereafter “distance,” between perceived risk and benefit (i.e., risk score minus benefit score). Consequently, riskier items of limited benefit receive higher distance scores compared to items judged as less risky and somewhat beneficial. Smaller distances show risks and benefits are judged similarly.

General linear models were fitted to investigate the relative importance of ecological and social factors in determining (a) the risk–benefit trade-offs (“distance”) of BG and WF; (b) the percentage of damage reduction wanted; and (c) the percentage of BG culling desired. Percentages of income derived from the farm and from conservation payments, valence, arousal, trust in local organizations, farm-specific goose density (BG and WF), farm-specific area of improved grassland and mean distance to roost (BG only) were all considered as fixed effects. Based on psychological and ecological rationale (Figure 1), for each model, 8–10 variables were selected that might influence the response variable (see Table 1). No co-occurring predictors were highly correlated (*r* > .70). We fitted models with all possible combinations of these variables as predictors, up to a maximum of four and five predictors in the management and risk–benefit models respectively, to avoid overfitting (Hair, Black, Babin, Anderson, & Tatham, 1998). We compared the parsimony of these models and reported standardized coefficient estimates from the best models (ΔAIC = 0), alongside conditional Akaike model averaged coefficients calculated across all fitted models to illustrate the level of consistency in coefficient estimates (Table 1). To explore the strength of evidence for these effects, we produced top model sets composed of supported models with ΔAIC ≤6 and lower than simpler nested models (Tables S5–S8; Richards, 2015).

## 3 Results

Between June and October 2016, most goose scheme-enrolled farmers (74%, *n* = 60) completed the questionnaire. Table S3 presents farm/farmer profiles. Most farmers (61.6%) were strongly supportive/supportive of the current goose scheme. Support was significantly lower (10.2% strongly supportive/supportive) under the scenario proposing payments were halved (Wilcoxon *T* = 144, *p* ≤ .001). When asked to design their own goose scheme, farmers wanted, on average, damage reduced by 39.2% (*SE* = 2.5) over 8.3 years (*SE* = 0.8), achieved by shooting 34.9% (*SE* = 2.4) of BG on Islay. There was consensus that money for goose payments should come from SNH via government funding (85.0%, *n* = 51). Few thought funds should come from the Scottish Rural Development Programme (21.6%, *n* = 13).

**Figure 2:**
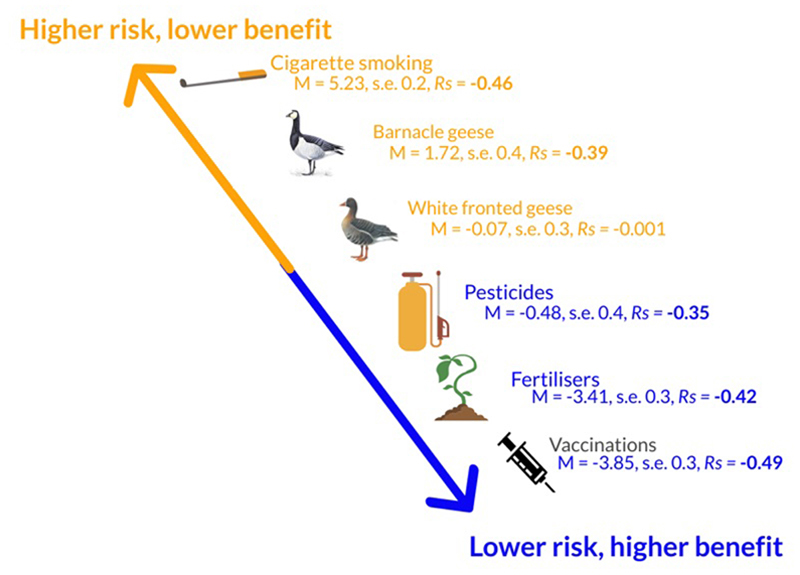
Mean distance scores calculated as risk score minus benefit score (*n* = 60). Riskier items of limited benefit receive higher distance scores compared to items judged as less risky and somewhat beneficial. Positive distance scores indicate risks outweigh benefits. The smaller the distance score, the greater the similarity between farmer's judgments of risks and benefits. Farmers were asked to think of risks/benefits broadly with respect to their livelihoods and household well-being. Risk/benefit assessment of lifestyle (e.g., smoking) and livelihood (e.g., fertilizers) items provide context. Spearman's Rank coefficients indicate the strength and direction of relationships between the perceived risks and benefits of each item; bold text indicates significance at the .01 level (2-tailed). Variable coding: Not at all risky/ beneficial = 1, neutral = 4, very risky/beneficial = 7. Goose images used with permission from the RSPB (Mike Langman [rspbimages.com])

Participants' trust in national and local branches of key organizations differed significantly (Wilcoxon NFU *T* = 530.5, *p* ≤ .001; RSPB *T* = 480.0, *p* ≤ .001; SNH *T* = 810.5, *p* ≤ .001); trust was consistently higher for local branches. Trust was similarly high for local-NFU (median = 5.0, IQR = 3) and local-SNH (median = 5.0, IQR = 2; Wilcoxon *T* = 606.0, *p* = .19), and was lower for local-RSPB (median 4.0, IQR = 3). With respect to their livelihoods and overall household well-being, farmers perceived smoking as the riskiest and least beneficial activity judged (Figure 2), followed by the presence of BG; the positive distance score for BG (1.72) indicates risks outweighed benefits. Vaccinations were viewed as the least risky/most beneficial item evaluated; the negative distance score (–3.85) shows benefits outweighed risks. Across all items, risks and benefits were negatively correlated.

**Figure 3:**
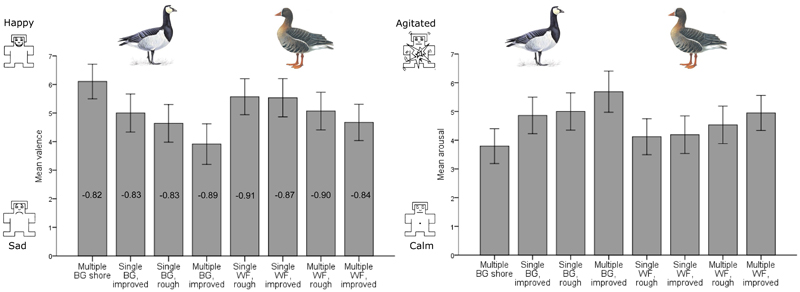
Mean valence and arousal judgements of photographs of single or multiple barnacle or white fronted geese in different habitats including the shore, rough or improved grazing. Error bars show 95% confidence intervals. Numbers beneath bars depict Spearman’s rank correlation coefficients identifying the strength and direction of relationships between valence and arousal for each goose image. All correlations are significant at the 0.01 level, (n=59). Goose images used with permission from the RSPB (Mike Langman [rspb-images.com]). Self-Assessment Manikin emojis (Bradley & Lang 1994)

Risk perceptions of geese were negatively and significantly related to valence and positively related to arousal; the direction of these relationships reversed for benefits (Table S4). The image of multiple BG on improved grassland received the lowest valence, and highest arousal score (Figure 3). Valence was negatively and significantly related to arousal across all goose stimuli; images stimulating an unhappy affective response, were judged as causing irritation (e.g., multiple BG on improved grassland *Rs* = –0.89, *p* ≤ .001). While valence is a feeling of pleasantness or unpleasantness, arousal reflects a subjective state of feeling deactivated or activated (Feldman Barrett & Russell, 1998), akin to feelings of irritation or agitation. Such feelings are frequently assumed to underpin retaliatory actions against problem-wildlife. Based on this premise, and to avoid issues of collinearity, we included arousal in models investigating goose management preferences and valence in models concerning goose risk perceptions.

**Figure 4:**
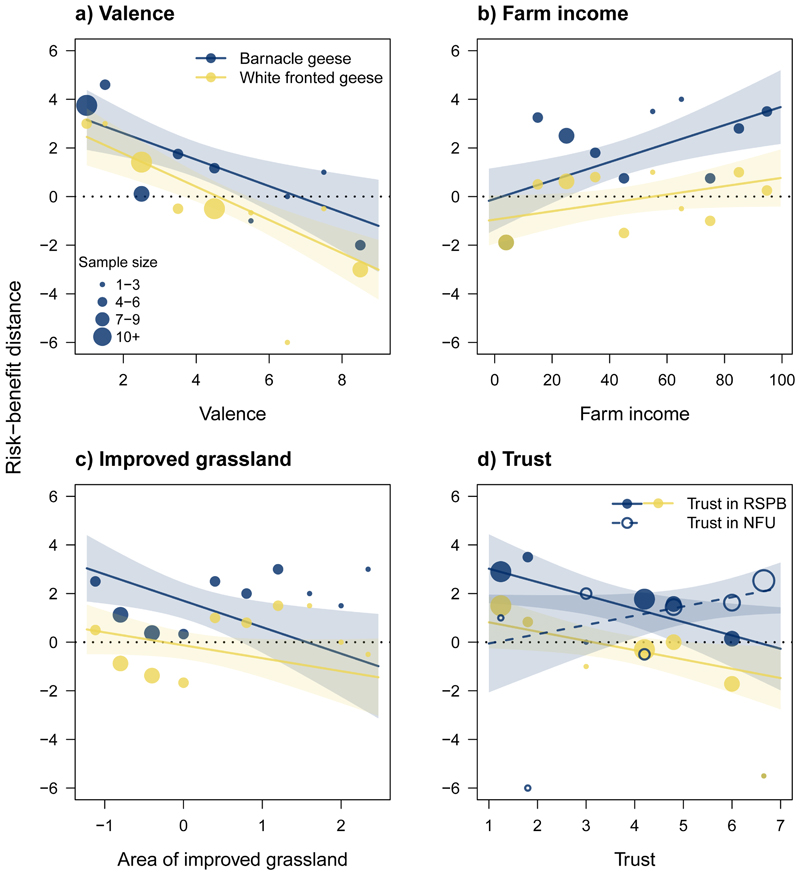
Fitted effects of social-ecological predictors on risk-benefit distance of barnacle goose (blue) and white-fronted goose (yellow): a) valence, b) percentage of income derived from farming, c) area of improved grassland, and d) trust in local organisations. Higher positive distance scores imply risks outweigh benefits, negative scores imply benefits outweigh risks. Lines indicate fitted estimates from the best performing models and shaded areas represent 95% confidence intervals around these estimates. Fitted values were calculated while setting other predictors to mean values. Points represent mean values calculated across different farmers for ten evenly spaced bands of each predictor. Point size indicates the number of farmers corresponding to each data point. In d) trust in RSPB is represented by solid points and lines; trust in NFU is represented by hollow points and dashed lines

As hypothesized, valence, trust in RSPB and area of improved grassland farmed were negatively related to farmers' risk perception of geese while trust in NFU and the proportion of household income derived from the farm were positively related to goose risk (Figure 4, Table 1). The best model explained 49% of the variance in farmers' risk perception of BG (*R* ^2^ = 0.49; Table S5). Risk “distance” of BG was strongly and negatively associated with farmer's affective response (valence) to the species (best model *β* = –0.44, confidence interval = –0.71, –0.16) and positively associated with farm income (best *β* = 0.36, CI = 0.13, 0.59); both occur in all top models — ΔAIC ≤6 and lower than simpler nested models (Figure 4a,b; Table 1 and Table S5). As valence increased, indicative of happiness, perceived risk declined whereas as household dependence on farm income increased, so too did the perceived risk of BG. The effect of improved grassland and trust in RSPB were moderate, each being retained in three of the top five models (grassland: best *β* = –0.33, CI = –0.60, –0.07; Trust_rspb_ best *β* = –0.31, CI = –0.57, –0.05). There was weak support for the influence of trust in NFU, being retained in one of the top models but being non-significant (best *β* = 0.21, CI = –0.03, 0.45) (Figure 4c,d; Table 1 and Table S5). There was no evidence for effects of trust in SNH, conservation income, BG density or distance to BG roost; for each of these predictors, model averaged effects were weak and nonsignificant (Table 1).

Retained in all top models, valence was also the strongest predictor of “distance” for WF (best *β* = –0.62, CI = –0.85, –0.38). There was evidence of a moderate effect of trust in local RSPB (best *β* = –0.26, CI = –0.50, –0.03) which was retained in three of the top five models. The influence of farm income (best *β* = 0.20, CI = –0.02, 0.42) and improved grassland were weaker (retained in three and one of the top models respectively) but non-significant (best *β* = –0.20, CI = –0.44, 0.04). The best model explained 53% of the variance in farmers' risk perception of WF (*R* ^2^ = 0.53); effect directions were as hypothesized and identified for BG (Figure 4a–d, Table 1 and Table S6). There was no evidence for effects of trust in SNH or NFU, conservation income or WF density on farmers' risk perceptions of WF; all these model averaged effects were weak and non-significant (Table 1).

**Figure 5:**
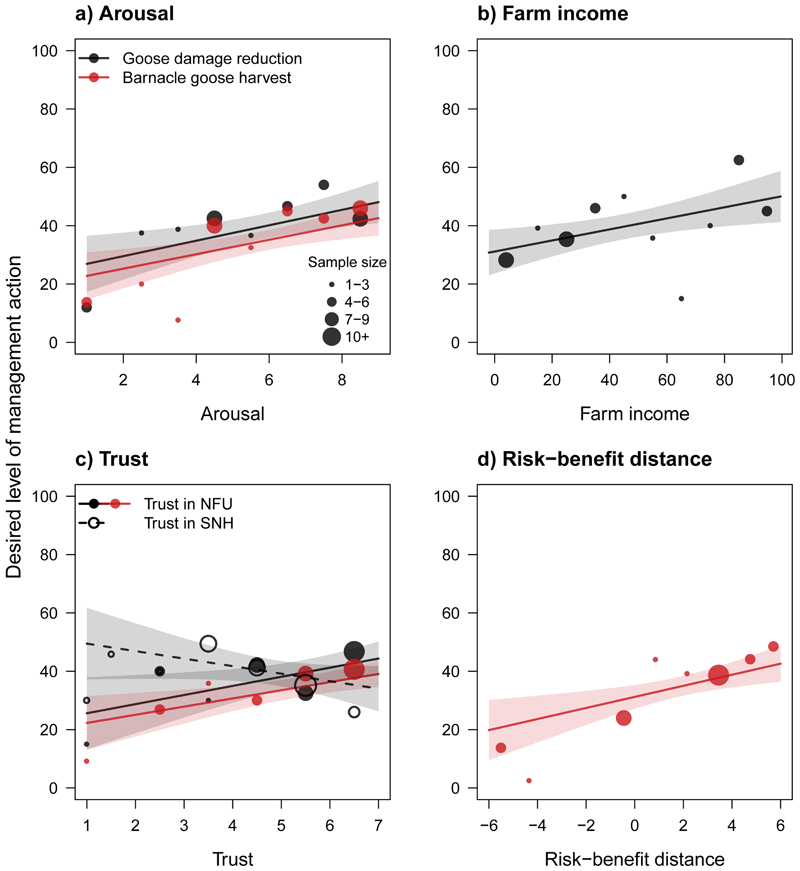
Fitted effects of social-ecological predictors on two aspects of barnacle goose management, damage reduction (black) and harvest (red): a) arousal, b) percentage of income derived from farming, c) trust in local organisations, and d) barnacle goose risk-benefit distance. Lines indicate fitted estimates from the best performing models and shaded areas represent 95% confidence intervals around these estimates. Fitted values were calculated while setting other predictors to mean values. Points represent mean values calculated across different farmers for ten evenly spaced bands in the predictor. Point size indicates the number of farmers corresponding to each data point. In c) trust in NFU is represented by solid points and lines; trust in SNH is represented by hollow points and dashed lines

As expected, arousal, trust in NFU and the proportion of household income derived from the farm were positively related to the severity of management farmers desired, while trust in SNH was weakly negatively related to the level of BG management farmers wanted to see (Figure 5, Table 2 and Table S7). The best model explained 49% of the variance in farmers' views regarding BG damage reduction (*R* ^2^ = 0.49; Table S7). Retained in all top models, arousal was the strongest predictor of the amount of BG damage reduction farmers wanted (best model *β* = 0.39, CI = 0.12, 0.65; Figure 5a, Table 2 and Table S7). As arousal increased, indicative of agitation, so too did the level of damage reduction preferred by farmers. Effect directions were the same for dependence on farm income which was retained in four of the top five models (best *β* = 0.34, CI = 0.09, 0.59) and trust in local-NFU retained in three of the top models (best *β* = 0.30, CI = 0.05, 0.55, Figure 4b,c, Table 2 and Table S7). The influence of trust in SNH was weaker and non-significant, being retained in two of the top models where it was negatively related to favored levels of damage reduction (best *β* = –0.22, CI = –0.48, 0.04). Affective responses to BG were also the strongest predictor of the percentage of BG farmers wanted shot (best *β* = 0.39, CI = 0.15, 0.63). As negative affect increased, so too did the percentage of BG they wanted culled (Figure 5a, Table 2 and Table S8). Trust in NFU (best *β* = 0.30, CI = 0.09, 0.50; Figure 4c) and risk–benefit “distance” (best *β* = 0.36, CI = 0.13, 0.60; Figure 5d) were also strongly and positively related to the proportion of BG farmers wanted culled. The best model explained 64% of the variance in farmers' desired level of BG harvest (*R* ^2^ = 0.64, Table S8).

## 4 Discussion

Evidence suggests people are not capable of divorcing the rational from the emotional when making decisions (Slagle et al., 2012; Slovic et al., 2007). We found strong relationships between affect and both risk perception and management preferences exist even for species that pose no direct danger to humans. Farmers' affective response to BG was the most important factor driving them to favor greater reductions in goose numbers and damage. However, our findings also revealed complexity in drivers of management preferences, with economic, social (trust in NFU) and risk factors having a significant positive influence on the desired reduction of BG and associated damage. The growing BG population, coupled with climate change exacerbates agricultural losses on Islay (Mason et al., 2018), the impact of which, to the household economy is substantial, particularly where farming is the primary source of income. Indeed, as dependence upon farm income increased, so too did the perceived risk of barnacle geese. In the same direction, increased trust in NFU meant farmers favored greater damage reduction and goose harvest. Trust is determined in part by value similarity (Zajac et al., 2012) and hazard acceptance theory stipulates perceived risks/benefits are a function of trust. Thus, greater trust in wildlife agencies theoretically leads to lower perceived risk (an effect we detected regarding RSPB) and greater wildlife acceptance (Bruskotter & Wilson, 2014; Siegrist, 2000). We observed that trust in RSPB was prominent in farmers' risk perception of geese (increased trust was associated with reduced risk) but not goose management; this likely reflects the prominence of RSPB in goose preservation as opposed to management (i.e., damage reduction on farms or culling). Our results show that trust in farming advocates (NFU) can be associated with preferences for more stringent wildlife management; this has substantial implications for the management of wildlife if we are to reverse farmland biodiversity loss and reconcile conservation and food production demands globally. Pre-emptive steps to reduce the quantity and severity of future conflicts might include striving for meaningful positive interactions and efforts for closer cooperation of policy makers, managers and conservation NGOs with the agricultural sector (Pollard et al., 2019).

Whereas risks and benefits are typically positively correlated in the world, evidence abounds that people perceive them as inversely correlated (Alhakami & Slovic, 1994; Slovic, Finucane, Peters, & MacGregor, 2004). This phenomenon is attributed to the “affect heuristic” whereby item evaluation is driven by rapid, automatic “feelings”—if an item is liked, it is simultaneously judged as high benefit, low risk (Finucane et al., 2000; Slovic et al., 2007). Farmers' evaluations of geese, lifestyle and livelihood actions in our study were no exception. This matters because social and political action to address specific risks, including ecosystem collapse and climate change, may be compelled or constrained by misguided public risk perceptions (Leiserowitz, 2006). While cigarette smoking was rated as the riskiest and least beneficial of the items scored, we revealed that farmers perceived the risk of BG to be higher than other threats including fertilizers and pesticides, the use of which they can control. The use of fertilizers and pesticides is accompanied by economic and health risks. However, in keeping with the land management demands of growing improved grassland on Islay, the benefits, particularly of fertilizers, were seen to outweigh their risks. Perceiving geese as risky/of little benefit may explain limits to goose conservation support. A study by Pollard et al. (2019) on greylag geese (*Anser anser*) in Scotland showed that intention of crofters to cooperate with management strategies was very high (>99%) but dropped considerably (to 77%) when there was administrative uncertainty that is, where scheme implementation was beyond their control.

Evidence of “risk as feelings” became evident in early psychometric studies of risk perception which revealed that the higher hazards scored on the “dread factor” (a composite score including perceived uncontrollability, dread, catastrophic potential), the riskier people perceive them to be (Slovic, 1987; Slovic et al., 2004). Subsequently, risk perceptions influenced the degree to which people wanted to see risk reduced via regulation (Slovic, 1987). Our findings affirm this logic, which we argue is particularly important when managing increasing wildlife populations. Islay's geese have increased 10-fold over the last 30–40 years and this increase has likely led to a sense of uncontrollability among farmers (Cusack et al., 2019). While people accept risks from voluntary activities (e.g., skiing) at levels around 1,000 times greater than involuntary risks (e.g., food preservatives) (Slovic, 1987), potential impacts of wildlife recovery represent involuntary risk to some stakeholders. Such uncontrollability may influence people's support for carnivores too, especially when species return after long absences and multiple rapidly (e.g., wolves in Germany; Arbieu et al., 2019). The effect of policies that protected BG from hunting, allowing the population to increase is evident; BG-related risk is now high among farmers, potentially hindering future conservation success. Differences in the degree of control farmers have over BG management may explain why we observed a relationship between risk and harvest but not risk and damage reduction. Farmers can implement BG damage reduction strategies (e.g., scaring) independently whereas culling is managed by SNH and is thus beyond their direct control. The uncontrollability of culling likely explains the strong association between risk and desired level of BG harvest.

Substantial effort is frequently invested in understanding the ecology of conflict systems. For example, analysis of historical spatial ecological and environmental data from Islay demonstrated that alongside the secondary role of climate change, habitat modification was the primary driver in the emergence of conflict between goose conservation and agriculture (Mason, Keane, et al., 2018). However, our models provide evidence of the prominence of human, as opposed to ecological characteristics, in driving species management decision-making by those impacted by wildlife. While the area of improved grassland managed by farmers was moderately associated with the perceived risk of BG, ecological factors including proximity to BG roost and farm-specific BG quantity, did not impact farmers' decision-making regarding goose management. Teixeira et al. (2020) similarly reported limited influence of landscape and species characteristics on Brazilian land-owners' tolerance for a range of species including opossum, crab-eating fox and puma.

Our research further highlights the potential of psychology to inform conservation (Eriksson et al., 2020; Papworth, 2017; Selinske et al., 2018). In contrast to previous research, we reveal how affect, risk and trust influence management preferences for specific levels of management desired (i.e., how many geese to cull), rather than general levels of support or opposition for different actions. Our study shows clear interactions between psychological (affect, trust and risk) and economic factors that need to be considered and managed if we are to halt the biodiversity crisis in human dominated landscapes. With respect to the “goose issue” on Islay, widening the discussion amongst stakeholders beyond damage reduction and financial compensation, to one that acknowledges the various emotions, both positive and negative, that BG give rise to amongst farmers, is a good place to start. Increasing the breadth of dialogue in such a way may bolster the effectiveness of the goose scheme as it currently operates. Such an intervention requires a neutral broker prepared to listen; in this respect, the local branch of SNH who currently administer the scheme, are strongly positioned to organize local working groups and workshops, potentially bringing in a professional mediator to allow the emotional side of the goose issue to be expressed and fed into management. The external, top-down decision-making design of Islay's goose scheme make it suitable for reducing human-wildlife impacts but weaker in terms of reducing the underlying human-human conflict (Redpath et al., 2013). Partly because of the use of culling as a BG management tool, it currently seems unlikely that key conservation organizations would be willing to enter dialogue, instead they continue to pursue an adversarial approach on banning culling. However, lobbying as a strategy to reduce culling can increase conflict between parties, leading to strong opposition which, at its most extreme, can lead to illegal harvesting which in turn can jeopardize conservation effectiveness (Cusack et al., 2020). On Islay and more broadly, management interventions that incorporate elements of the less “rational” human, including factors such as affect, trust, norms, identity and history, into their design may be more effective in securing ongoing engagement (Pooley, 2016; St. John, Steadman, Austen, & Redpath, 2019). In 2019, the United Nations launched the Decade for Ecosystem Restoration (United Nations, 2019), signed by over 70 countries worldwide. The success of this resolution will not only depend on actions towards restoring ecosystems and species, but also on gaining people's support, mitigating conservation conflicts and the legacy of conservation success.

## Supplementary Material

Questionnaire

Supporting Information

## Figures and Tables

**Table 1:** Standardized linear coefficients and confidence intervals from models of risk–benefit distance

Predictor	Risk–benefit distance BG		Risk–benefit distance WF	
	Best *β*	Average *β*	Best *β*	Average *β*
Valence_BG_	**–0.44 (–0.71, –0.16)**	**–0.50 (–0.82, –0.18)**	n/a	n/a
Valence_WF_	n/a	n/a	**–0.62 (–0.85, –0.38)**	**–0.62 (–0.90, –0.34)**
Trust_SNH_	—	0.04 (–0.27, 0.36)	—	–0.03 (–0.31, 0.26)
Trust_NFU_	0.21 (–0.03, 0.45)	0.18 (–0.09, 0.45)	—	–0.02 (–0.26, 0.22)
Trust_RSPB_	**–0.31 (–0.57, –0.05)**	–0.28 (–0.57, 0.01)	**–0.26 (–0.50, –0.03)**	-0.25 (-0.51, 0.01)
Farm income	**0.36 (0.13, 0.59)**	**0.34 (0.09, 0.59)**	0.20 (–0.02, 0.42)	0.19 (–0.04, 0.42)
Cons. income	—	0.13 (–0.16, 0.41)	—	0.09 (–0.19, 0.37)
Density_BG_	—	0.03 (–0.25, 0.31)	n/a	n/a
Density_WF_	n/a	n/a	—	0.07 (–0.16, 0.30)
Dist. to roost	—	–0.04 (–0.30, 0.22)	n/a	n/a
Improved grass	**–0.33 (–0.60, –0.07)**	**–0.30 (–0.60, –0.01)**	–0.20 (–0.44, 0.04)	–0.18 (–0.45, 0.09)

*Note:* Coefficients from top performing models (ΔAIC = 0) and conditional Akaike model-averaged coefficients calculated across all fitted models are provided. For each model, we considered all combinations of 8–10 potential predictor variables, up to a maximum of five predictors per model. Bold indicates significance at *p* < .05.

**Table 2:** Standardized linear coefficients and confidence intervals from models of desired barnacle goose damage reduction and harvest

Predictor	Damage reduction		Harvest	
	Best *β*	Average *β*	Best *β*	Average *β*
Risk-benefit_BG_	—	0.09 (–0.33, 0.50)	**0.36 (0.13, 0.60)**	**0.38 (0.11, 0.65)**
Arousal_BG_	**0.39 (0.12, 0.65)**	**0.44 (0.13, 0.74)**	**0.39 (0.15, 0.63)**	**0.42 (0.13, 0.71)**
Trust_SNH_	–0.22 (–0.48, 0.04)	–0.22 (–0.52, 0.08)	—	0.05 (–0.18, 0.28)
Trust_NFU_	**0.30 (0.05, 0.55)**	**0.29 (0.01, 0.57)**	**0.30 (0.09, 0.50)**	**0.30 (0.08, 0.52)**
Trust_RSPB_	—	–0.09 (–0.50, 0.31)	—	0.10 (–0.18, 0.39)
Farm income	**0.34 (0.09, 0.59)**	**0.31 (0.04, 0.60)**	—	0.07 (–0.18, 0.31)
Cons. income	—	–0.06 (–0.38, 0.25)	—	–0.03 (–0.27, 0.21)
Density_BG_	—	–0.01 (–0.37, 0.34)	—	–0.05 (–0.31, 0.20)
Dist. to roost	—	–0.10 (–0.40, 0.20)	—	0.05 (0.17, 0.28)
Improved grass	—	0.18 (–0.16, 0.52)	—	0.09 (–0.19, 0.37)

*Note:* Coefficients from top performing models (ΔAIC = 0) and conditional Akaike model-averaged coefficients calculated across all fitted models are provided. For each model, we considered all combinations of 8–10 potential predictor variables, up to a maximum of four predictors per model. Bold indicates significance at *p* < .05.

## Data Availability

The anonymized data used in this study will be made freely available upon request.
