# Supplementary material for: The role of risk perception and affect in predicting support for conservation policy under rapid ecosystem change
Source: Conserv Sci Pract. Author manuscript; Available in PMC 2021 Mar 1. (PMC7116843; doi:10.1111/csp2.316)
Supplement: Questionnaire [file EMS116186-supplement-Questionnaire.pdf]

I am a researcher from the University of Kent and I am conducting a study concerning how people on Islay view geese. So that we collect data from as many people as possible, I would be grateful if you would take time to complete this questionnaire. Anonymised data from this study will be useful in discussions of goose management strategies and will be used to write a scientific publication. Before you decide to participate, please could you confirm if you consent to completing this questionnaire and the use of your response for the above purposes?

Agree *(please tick)*		Disagree		Date:

About your farm:		Hectares
1	Total size of farm	
2	Area of improved grassland (i.e. grassland reseeded in the last 7 years)	
3	Area used for rotational arable cropping	
4	Area permanent pasture (pasture 8 years or older)	
5	Other *(please specify type & size)*<br>*Note: sum of land uses should equal 'Total size of farm'*	

LIVESTOCK		Quantity *(enter quantity e.g. 0, 1, 2…)*
6	How many beef cattle do you currently stock?	
7	How many sheep do you currently stock?	
8	Other livestock *(specify type & quantity)*	
9	What year did you start receiving goose payments? *(provide year)*:	

10	What is your year of birth *(e.g. 1974)*	
11	What is your gender? *(Circle one)*	Male Female
12	Which of the following best describes your situation with this farm? *(Circle one)*<br>Other: ______________________________	Owner<br>Tenant farmer<br>Crofter
13	How long have you managed this farm for? *(In years)*	
14	How long have you been farming for in total? *(In years)*	

I would now like to present some ***scenarios*** to you about the future of farming and barnacle goose management on Islay. I'm going to ask you to report your level of support for the scenario using this scale *[show 'scenario' scale]*

Scenario A

Over a 10 year period goose damage will be reduced by 25-35%. This will be achieved by shooting 25-30% of the barnacle geese on Islay over 10 years. Goose payments will remain the same. Subsidies and average prices of agricultural goods (both purchased and sold) will remain at today's levels, although some small variation can be expected between years.

**A**	Please indicate your level of support for the above scenario: *(please circle one)*			
Very unsupportive	Unsupportive	Neither supportive or unsupportive	Supportive	Strongly supportive

Why did you give this answer? ____

____

____

Scenario B

Over a ____ year period goose damage will be reduced by ____ - ____%. This will be achieved by shooting ____ - ____% of the barnacle geese on Islay over ____ years. Goose payments will remain the same. Subsidies and average prices of agricultural goods (both purchased and sold) will remain at today's levels, although some small variation can be expected between years.

**B**	Please indicate your level of support for the above scenario: *(please circle one)*			
Very unsupportive	Unsupportive	Neither supportive or unsupportive	Supportive	Strongly supportive

Why did you give this answer? ____

____

____

Scenario C

Over a 10 year period you and other Islay farmers double the number of sheep you stock to approximately 2/ha. As a result, barnacle geese on Islay will move elsewhere, for example, to other islands and goose damage will be reduced by 25-35% without shooting geese. Goose payments will remain the same. Subsidies and average prices of agricultural goods (both purchased and sold) will remain at today's levels, although some small variation can be expected between years.

**C**	Please indicate your level of support for the above scenario: *(please circle one)*			
Very unsupportive	Unsupportive	Neither supportive or unsupportive	Supportive	Strongly supportive

Why did you give this answer? ____

____

____

Scenario D

Over a 10 year period goose damage will be reduced by 25-35%. This will be achieved by shooting 25-30% of the barnacle geese on Islay over 10 years. Goose payments will reduce to half their current value due to funding cuts. Subsidies and average prices of agricultural goods (both purchased and sold) will remain at today's levels, although some small variation can be expected between years.

**D**	Please indicate your level of support for the above scenario: *(please circle one)*			
Very unsupportive	Unsupportive	Neither supportive or unsupportive	Supportive	Strongly supportive

Why did you give this answer? ____

____

____

In ***general***, how risky do you consider each of the following items to be for your livelihood and overall household wellbeing? Think about risk in broad terms.								
	**RISK SCALE**	**1**	**2**	**3**	**4**	**5**	**6**	**7**
15	Chemical fertilisers							
16	Cigarette smoking							
17	Barnacle geese							
18	Pesticides							
19	Vaccinations							
20	White fronted geese							

Please tell me how much you agree or disagree with the following statements:							
	(Tick one box for each statement)	Strongly agree	Agee	Neither agree nor disagree	Disagree	Strongly disagree	Don't know
21	Money for goose payments should come from Scottish National Heritage/Scottish Government						
22	Money for goose payments should come from the Scottish Rural Development Programme						

In ***general***, how beneficial do you consider each of the following items to be for your livelihood and overall household wellbeing? Think about benefits in broad terms.								
	**BENEFIT SCALE**	**1**	**2**	**3**	**4**	**5**	**6**	**7**
23	Chemical fertilisers							
24	Cigarette smoking							
25	Barnacle geese							
26	Pesticides							
27	Vaccinations							
28	White fronted geese							

I am going to show you some images and using two different scales, I would like you to rate each picture in terms of how it made you feel while viewing it *[show & explain scales].*

Some of the pictures may prompt emotional experiences; others may seem relatively neutral. Your rating of each picture should reflect your immediate personal experience, and no more. Please rate each one as up actually felt while you watched the picture. There are no right or wrong answers, so simply respond as honestly as you can. You will see each image for 6 seconds. I will start with an example *[sheep example]*

	**Image (A=Valence, B=Arousal)**	**1**	**2**	**3**	**4**	**5**	**6**	**7**	**8**	**9**
29	BG improved grass land (A)									
30	BG improved grass land (B)									
31	Zip (A)									
32	BG flock imp. Grassland (A)									
33	BG flock imp. Grassland (B)									
34	Hospital (A)									
35	BG natural habitat (A)									
36	BG natural habitat (B)									
37	Children (A)									
38	BG flock in natural habitat (A)									
39	BG flock in natural habitat (B)									

In general, how much trust do you have in the following organisation to make balanced decisions about land and wildlife management on Islay? I will first ask you about the national office of the organisation & then the local office.								
	**TRUST SCALE**	**1**	**2**	**3**	**4**	**5**	**6**	**7**
40	National Farmers Union							
41	RSPB							
42	Scottish Government, Rural Payments & Inspections Directorate							
43	Scottish Natural Heritage (SNH)							

I am going to show you some more images and using two different scales, I would like you to rate each picture in terms of how it made you feel while viewing it *[provide scales A & B].*

	**Image (A=Valence, B=Arousal)**	**1**	**2**	**3**	**4**	**5**	**6**	**7**	**8**	**9**
44	WF improved grass land (A)									
45	WF improved grass land (B)									
46	Rat (A)									
47	WF flock imp. Grassland (A)									
48	WF flock imp. Grassland (B)									
49	Sea (A)									
50	WF natural habitat (A)									
51	WF natural habitat (B)									
52	Girl (A)									
53	WF flock in natural habitat (A)									
54	WF flock in natural habitat (B)									

	By what name do you know the bird shown in the image? *(write name in full)*	
55	Image A	
56	Image B	

	Thinking in terms of rough percentages (not actual amounts of cash), what proportion of your family income is derived from:	Estimated percentage (%)
57	Farming activities	
58	Conservation agreements (e.g. goose payments)	
59	Letting of holiday accommodation	*(If % go to 62)*
60	Off-farm employment	

	What percentage of your holiday lettings are accounted for by:	Estimated percentage (%)
61	Whisky tourists	
62	Bird-watching/wildlife tourists	
63	Other 'general' tourists	

Do you mind if I record your address on this questionnaire? This would enable me to conduct spatial analyses, for example examining how distance from goose roosting sites affects you & your farming. I will never identify you or your farm when analysing and presenting the data.
Name & address *(Optional)*:
