# Supplementary material for: The role of risk perception and affect in predicting support for conservation policy under rapid ecosystem change
Source: Conserv Sci Pract. Author manuscript; Available in PMC 2021 Mar 1. (PMC7116843; doi:10.1111/csp2.316)
Supplement: Supporting Information [file EMS116186-supplement-Supporting_Information.pdf]

Supporting Information

F.A.V. St. John, Mason, T.H.E., Bunnefeld, N., *The role of risk perception and affect in predicting support for conservation policy under rapid ecosystem change.* Conservation Science and Practice.

Table S1 Images of barnacle geese and white fronted assessed by farmers using the valence and arousal scales. Positive valence, Happy, Pleased = 9; negative valence, Unhappy, Annoyed = 1. Positive arousal, Calm, Relaxed = 1; negative arousal, Agitated, Irritated = 9. Photographs were professionally printed, size 17.8 cm x 12.7 cm.

Branta leucopsis	*Anser albifrons flavirostris*	
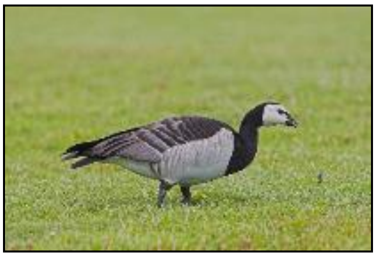	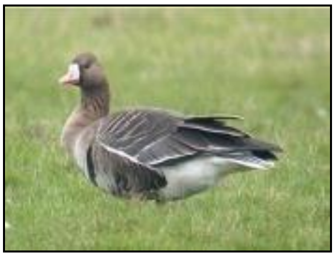	Single goose on improved grassland
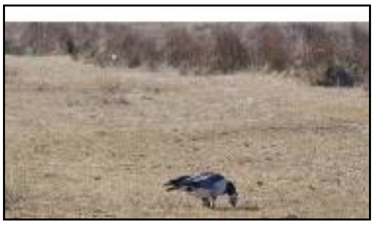	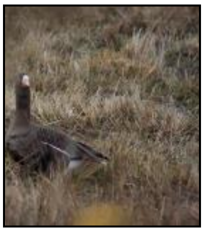	Single goose on natural habitat
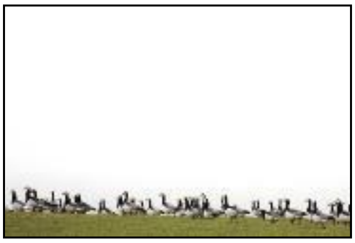	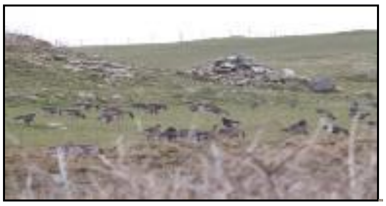	Multiple geese on improved grassland
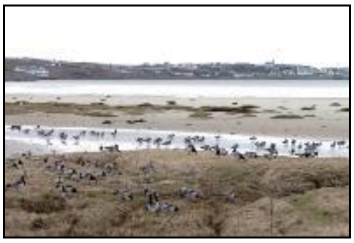	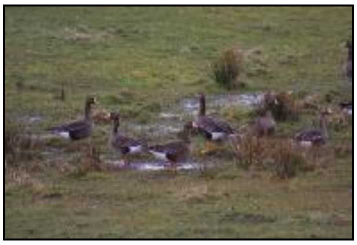	Multiple geese on natural habitat

Table S2 Mean (standard deviation) valence scores reported for six images from the IAPS library. Valence scores from our 60 study participants are presented next to those of 50 male college students reported in Lang et al. (2008). IAPS images cannot be printed in publications due to continued use in studies.

	Mean (std dev) valence reported by farmers[1]	Mean (StDev) valence reported by 50 male American college students (Lang et al. 2008)[1]
IAPS 7045, zipper	5.51 (1.71)	5.14 (0.43)
IAPS 3220, hospital	2.80 (1.80)	2.59 (1.28)
IAPS 2216, kids	8.29 (1.46)	7.12 (1.41)
IAPS 1280, rat	1.57 (1.33)	4.40 (1.70)
IAPS 5825, sea	8.33 (1.19)	8.08 (0.86)
IAPS 2411, girl	6.22 (2.05)	5.10 (0.80)

[1]Positive valence, Happy, Pleased = 9; negative valence, Unhappy, Annoyed = 1.

Table S3 Farmer demographic and farm profile

Description	Value (SE)
Sex	88.3% males
Mean age (n=58)	63 (1.6)
Farm details (n=58):	
Mean farm size	338.7 ha (56.4)
Mean area of improved grassland	53.7 ha (5.7)
Household income (n=55):	
Mean percentage of household income derived from:	
Farming	46.2% (4.4)
Off-farm employment	36.3% (4.8)
Conservation payments	9.4% (1.6)
Letting holiday accommodation	8.1% (2.5)
Percentage of farmers letting holiday accommodation (n=19)	31.7%
Mean percentage of household income derived from:	
General tourism including game-shooting	51.5% (7.8)
Whiskey-related tourism	33.4% (6.6)
Wildlife viewing tourism	15.2% (4.0)

Table S4 Spearman's rank correlations between risk, valence and arousal, and benefits, valence and arousal (n=59). Bold text indicates significance at 0.01 level; italic at 0.05 level.

Item	Risk[1]: Valence[2]	Risk: Arousal[3]	Benefit[4]: Valence	Benefit: Arousal
Barnacle goose				
Single BG, improved grass	*-0.32*	*0.32*	*0.33*	*-0.32*
Multiple BG, improved grass	**-0.38**	**0.36**	**0.36**	*-0.43*
Single BG, rough grazing	-0.19	0.14	0.11	*-0.12*
Multiple BG, shore grazing	**-0.33**	*0.50*	*0.34*	*-0.33*
White fronted goose				
Single WF, improved grass	**-0.62**	**0.60**	*0.32*	**-0.37**
Multiple WF improved grass	**-0.53**	**0.50**	*0.32*	*-0.26*
Single WF rough	**-0.63**	**0.61**	0.26	*-0.30*
Multiple WF rough	**-0.56**	**0.50**	**0.25**	*-0.28*

[1] Not at all risky = 1, Very risky =7

[2] Valence: Happy/Pleased =9, Unhappy/Annoyed =1

[3] Arousal: Calm/Relaxed = 1, Agitated/Irritated =9

[4] Not at all beneficial =1, Very beneficial =7

Table S5 Standardised linear coefficients and performance statistics of best performing models of barnacle goose risk-benefit distance. All combinations of nine predictor variables were considered, up to a maximum of five predictors per model. Models with ΔAIC ≤ 6 and lower than simpler nested models were retained (Richards, 2015). The number of parameters in each model (*K*), log-likelihood (LL), ΔAIC and R^2 are shown.

Valence, BG	Proportion of income from farm	Area improved grass	Trust, NFU local	Trust, RSPB local	*K*	LL	ΔAIC	R^2
-0.44	0.36	-0.33	0.21	-0.31	7	-50.48	0.00	0.49
-0.50	0.35	-0.29		-0.25	6	-52.08	1.21	0.45
-0.59	0.37	-0.25			5	-54.11	3.26	0.40
-0.40	0.29			-0.20	5	-54.61	4.27	0.39
-0.48	0.31				4	-55.84	4.73	0.36

Table S6 Standardised linear coefficients and performance statistics of best performing models of white-fronted goose risk-benefit distance. All combinations of eight predictor variables were considered, up to a maximum of five predictors per model. Models with ΔAIC ≤ 6 and lower than simpler nested models were retained (Richards 2015). The number of parameters in each model (*K*), log-likelihood (LL), ΔAIC and R^2 are shown.

Valence, WF	Proportion of income from farm	Area improved grass	Trust, RSPB local	*K*	LL	ΔAIC	R^2
-0.62	0.20	-0.20	-0.26	6	-48.41	0.00	0.53
-0.56	0.15		-0.22	5	-49.89	0.95	0.50
-0.55			-0.25	4	-50.96	1.09	0.48
-0.65	0.19			4	-51.66	2.49	0.46
-0.65				3	-53.13	3.43	0.43

Table S7 Standardised linear coefficients and performance statistics of best performing models of desired reduction in barnacle goose damage. All combinations of ten predictor variables were considered, up to a maximum of five predictors per model. Models with ΔAIC ≤ 6 and lower than simpler nested models were retained (Richards 2015). The number of parameters in each model (*K*), log-likelihood (LL), ΔAIC and R^2 are shown.

Arousal, BG	Proportion of income from farm	Trust, NFU local	Trust, SNH local	*K*	LL	ΔAIC	R^2
0.39	0.34	0.30	-0.22	6	-40.53	0.00	0.49
0.46	0.31	0.28		5	-42.10	1.15	0.45
0.44	0.32		-0.19	5	-43.51	3.96	0.41
0.50	0.30			4	-44.52	3.99	0.37
0.50		0.26		4	-45.05	5.04	0.36

Table S8 Standardised linear coefficients and performance statistics of best performing models of desired barnacle goose harvest. All combinations of ten predictor variables were considered, up to a maximum of five predictors per model. Models with ΔAIC ≤ 6 and lower than simpler nested models were retained (Richards 2015). The number of parameters in each model (*K*), log-likelihood (LL), ΔAIC and R^2 are shown.

Arousal, BG	Risk-benefit distance, BG	Trust, NFU local	*K*	LL	ΔAIC	R^2
0.39	0.36	0.30	5	-35.91	0.00	0.64

References

Lang, P.J., Bradley, M.M. & Cuthbert, B.N. (2008). *International affective picture system (IAPS): Affective ratings of pictures and instruction manual. Technical Report A-8*. Gainesville, FL.

Richards, S.A. (2015). Likelihood and model selection. In: *Ecol. Stat. Contemp. theory Appl.* (eds. Fox, G., Negrete-Yankelevich, S. & Sosa, V.). Oxford University Press, Oxford, UK., pp. 58–78.
